# Cerebral venous sinus thrombosis presenting with multifocal intracerebral hemorrhage and subarachnoid hemorrhage: A case report

**DOI:** 10.1097/MD.0000000000013476

**Published:** 2018-12-14

**Authors:** Jing Sun, Zhidong He, Guangxian Nan

**Affiliations:** a Department of Neurology; b Department of Neurosurgery, China-Japan Union Hospital of Jilin University, No. 126 Xiantai Street, Changchun, Jilin 130000, China.

**Keywords:** cerebral venous sinus thrombosis, multifocal hemorrhage, subarachnoid hemorrhage

## Abstract

**Rationale:**

Multifocal cerebral hemorrhage refers to the cerebral hemorrhage in 2 or more lesions at the same time or 48 h in the brain caused by various causes, which has an acute onset, high mortality rate, and poor clinical treatment effect. Subarachnoid hemorrhage (SAH) is caused by the direct flow of blood into the subarachnoid cavity due to the rupture of the diseased vessels at the base or surface of the brain. Cerebral venous sinus thrombosis (CVST) affects approximately 5 people per million and accounts for approximately 1% of all stroke events. CVST with both SAH and multifocal intracerebral hemorrhage (ICH) as the first presentation is extremely rare.

**Patient concerns:**

A 57-year-old woman presented with dizziness, nausea, and vomiting.

**Diagnosis:**

Neuroimaging confirmed a diagnosis of CVST.

**Interventions:**

The patient was treated with dehydration, scavenging free radicals, and nerve protection therapy.

**Outcomes:**

After 4 weeks of systematic treatment, the patient resumed independent daily activities and was discharged with only slight non-fluent aphasia. She did not exhibit recurrent thrombosis at an 18-month follow-up point.

**Main Lessons:**

The usual treatment for sinus thrombosis is anticoagulation or local thrombolysis. Systemic anticoagulation is the first-line treatment for CVST, even in patients with cerebral hemorrhage or SAH. The present patient's hemorrhage clearly contraindicated heparin; therefore, no anticoagulants or thrombolytic agents were administered during the 4-week hospitalization. We discuss issues for consideration in similar cases and provide an example of determining an individualized approach to treatment.

## 1 Introduction

Cerebral venous sinus thrombosis (CVST) is a rare special cerebrovascular disease, accounting for 1% of all strokes, but the mortality rate is as high as 10%.^[1,2]^ There are many causes and risk factors of CVST, such as pregnancy, oral contraceptive, infection, trauma, central nervous system tumor, and coagulation dysfunction diseases. Initial presentation with subarachnoid hemorrhage (SAH) and intracerebral hemorrhage (ICH) are rare but can further complicate treatment and prognosis. Here we describe the case of a 57-year-old woman with interior and superior sagittal sinus thrombosis, who presented with SAH and multifocal hemorrhage as the first manifestations. The value of early diagnosis using computed tomography (CT) and magnetic resonance venography (MRV) is emphasized because it is important to identify possible causes of symptoms; it may be possible to reverse symptoms with appropriate individualized treatments.

## 2 Case report

**Figure 1:**
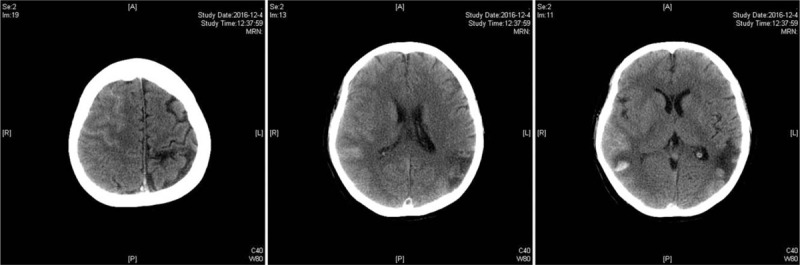
On admission, a CT scan revealed linear high-density images in the partial gutter of the right frontal, parietal, and occipital lobes, as well as patchy high-density images in the right parietal and left temporal lobes. CT = computed tomography.

**Figure 2:**
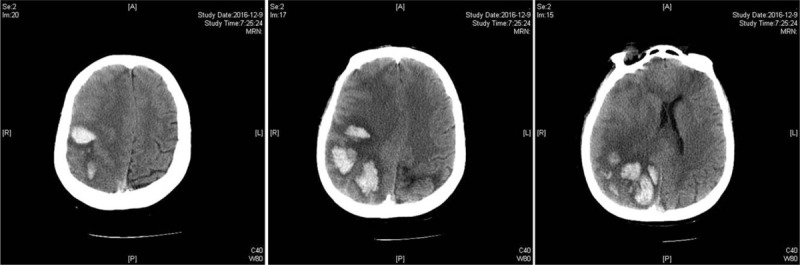
On the fourth day of hospitalization, CT reexamination demonstrated multiple patchy high-density shadows in the right occipital and parietal lobes. CT = computed tomography.

**Figure 3:**
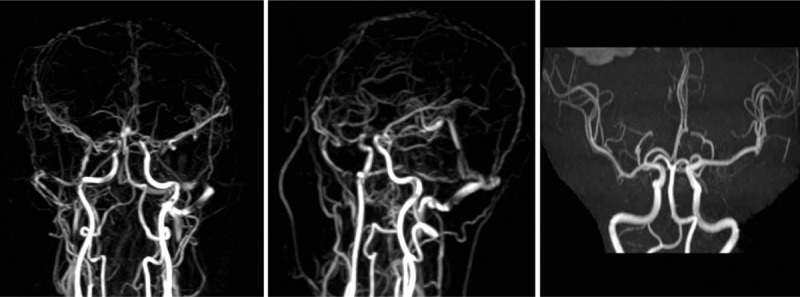
On the fourth day of hospitalization, MRA was normal, whereas MRV revealed interior and superior sagittal sinus thrombosis. MRA = magnetic resonance angiography; MRV = magnetic resonance venography.

The patient was admitted to our hospital with complaints of dizziness, nausea, and vomiting. She had a clinical history of the patent oval foramen and ischemic stroke with residual glossolalia. She denied any history of diabetes, hypertension, major surgery, trauma, nonsteroidal anti-inflammatory drug use, or illicit drug use. On admission, she was afebrile with blood pressure of 121/85 mmHg and heartbeat of 63/min. Neurological examinations revealed incomplete motor aphasia, nystagmus, and right hemihypalgesia. A CT scan demonstrated linear high-density images in the partial gutter of the right frontal, parietal, and occipital lobes, as well as patchy high-density images in the right parietal lobe and left temporal lobe (Fig. 1). Pertinent laboratory findings at the time of initial presentation revealed that cardiac troponin, coagulation, homocysteine, and ions, as well as renal and liver function, were normal. Additionally, the patient's white blood cell count was 10.22 thousand/cu mm. On the fourth day of hospitalization, the patient experienced an epileptic seizure. CT re-examination demonstrated that the right occipital and parietal lobes exhibited multiple patchy high-density shadows (Fig. 2). Magnetic resonance angiography (MRA) and MRV confirmed the CT findings and revealed interior and superior sagittal sinus thrombosis (Fig. 3). However, both tests were negative for aneurysms or other vascular malformations. The patient was diagnosed with CVST and was treated with dehydration, scavenging free radicals, and nerve protection therapy. No anticoagulants or thrombolytic agents were administered during the 4-week hospitalization. At discharge, the patient could complete independent daily activities and had only slight non-fluent aphasia. Follow-up brain CT at 12 weeks after onset demonstrated encephalomalacia in the right frontal, temporal, and parietal lobes, as well as in both the left temporal and occipital lobes. During the 18-month follow-up, the patient did not experience recurrent thrombosis, the symptoms of non-fluent aphasia were improved but did not return to normal, and the right limb muscle strength was slightly worse than normal, without sensory disorder.

The study was approved by the Institutional Review Board and Ethics Committee of China-Japan Union Hospital of Jilin University. The patient provided written informed consent for publication of this case report.

## 3 Discussion

CVST occurs when a blood clot forms in any of the venous sinuses of the brain. Approximately 5 people per million are affected by CVST and it accounts for approximately 1% of all stroke events.^[1]^ Although CVST is observed with increasing frequency in daily practice and has a variety of nonspecific clinical symptoms that overlap with other disorders, its presentation with an associated SAH or ICH via CT and magnetic resonance imaging (MRI) is infrequent. However, SAH is becoming more frequently recognized as a potential complication of CVST. Panda et al^[3]^ reported that 10 (4.3%) of 233 patients with CVST exhibited evidence of cortical SAH. Furthermore, Oda et al^[4]^ found that CVST resulted in 3% of SAH in a retrospective review, indicating that the presence of cortical SAH without involvement of the basal cisterns may be an early indicator of underlying CVST.^[3,4]^ However, the co-occurrence of SAH and multifocal hemorrhage with CVST has not previously been described.

The distribution of SAH associated with CVST typically differs from the characteristic pattern of SAH with arterial origins. Specifically, when SAH is localized at the cerebral convexity and spares the basal cisterns and skull base, CVST should be considered. The exact mechanism of cortical CVST-induced SAH is unknown, although rupture of venous parenchymal hemorrhagic infarcts into the subarachnoid space is a possibility.^[5,6]^ This mechanism is potentially applicable to the current patient because signs of multifocal hemorrhage were observed on her CT scans. Another possible mechanism is venous hypertension and subsequent rupture of dilated, thin-walled, bridging subarachnoid cortical veins devoid of smooth muscle fibers.^[5]^ In these cases, SAH typically occurs in the region adjacent to thromboses in veins or sinuses. This second mechanism may have also occurred in our patient, who had bleeding in the partial gutter of the right frontal, parietal, and occipital lobes, adjacent to the superior sagittal sinus.

In patients with CVST, spontaneous intracranial hemorrhage accounted for 30% to 40% of ICH.^[7]^ CVST-induced ICH includes simple cerebral hemorrhage and venous infarction hemorrhage. Furthermore, the distribution of venous infarction hemorrhages typically does not conform to the normal distribution of simple cerebral hemorrhage; the hematoma usually occurs closer to the surface of the brain, with a large area of low density around the focal point. Intramedullary or subcortical meniscus hemorrhage is the earliest manifestation of CVST combined with ICH, and the "zebra striated” hemorrhage is also a common characteristic of this combination. In the present case, bleeding in the right occipital and parietal lobes was considered a venous infarction hemorrhage, which may be attributed to the blockade of venous sinuses. Increased venous and capillary pressure results in diapedesis of red blood cells and subsequent rupture of small vessels. Therefore, ICH may be an extension of this sequence of events.

Because thrombosis is typically treated with anticoagulation or local thrombolysis. Systemic anticoagulation is the first-line treatment for CVST, due to high efficacy, safety, and feasibility, even in patients with cerebral hemorrhage or SAH. The 2011 American Heart Association/American Stroke Association guidelines recommend that once imaging confirms CVST, anticoagulation should be initiated regardless of whether ICH is evident at presentation.^[1]^ However, some patients with CVS and ICH present a quintessential example of the controversy regarding anticoagulation treatment,^[8]^ thereby emphasizing a careful consideration of the risks and benefits of anticoagulation in patients with combined CVST and ICH. The patient in the present study had SAH and multifocal hemorrhage; therefore, the extension and severity of the bleed indicated that anticoagulation should not be initiated. Fortunately, the patient experienced successful recovery after 4 weeks of systematic treatment. There are no specific recommendations about the timing of anticoagulation in CVST patients with ICH.^[9]^ Considering that this patient has many cerebral hemorrhage lesions, we recommend the patient take oral anticoagulant drugs after the absorption of intracranial hemorrhage, but the patient and her family refused.

## 4 Conclusion

Cerebral venous thrombosis rarely presents as acute SAH, and the clinical presentation may mimic an aneurysmal bleed. CVST should be considered in the differential diagnosis of patients presenting with SAH without evidence of an aneurysm. The radiologist plays an essential role in making this often-unsuspected critical diagnosis to enable prompt and appropriate treatment.

While anticoagulants are recommended in theory for treatment of cerebral sinus thrombosis, the present case report illustrates the imperativeness of individualized therapy. Similarly, intracerebral hematomas should theoretically be evacuated, although individualized therapy is critical. The present patient's hemorrhage was a clear contraindication to heparin. We recognize that successful neurological recovery was certainly not guaranteed in the present patient, nor would the same management work in every case; rather, this case illustrates the issues that must be considered in similar cases and provides an example of determining an individualized approach to treatment.

## Author contributions

**Data curation:** Zhidong He.

**Supervision:** Guangxian Nan.

**Writing – original draft:** Jing Sun.

**Writing – review and editing:** Jing Sun.
